# Investigating the influence of partner attitudes, norms and risk behavior on condom use decision-making during penile-vaginal sex with casual partners: a vignette study among dutch young people

**DOI:** 10.1186/s12889-025-24741-6

**Published:** 2025-10-08

**Authors:** Alcira de Vries, Janneke C.M. Heijne, John B.F. de Wit, Chantal den Daas

**Affiliations:** 1 Centre for Infectious Disease Control, National Institute for Public Health and the Environment, P.O. Box 1, Bilthoven, 3720 BA the Netherlands; 2 Department of Infectious Diseases, Public Health Service of Amsterdam, Amsterdam, The Netherlands; 3 Amsterdam institute for Immunology & Infectious Diseases (AII), Amsterdam Public Health research institute (APH), Amsterdam UMC Locatie AMC, Amsterdam, The Netherlands; 4 Department of Interdisciplinary Social Science, Utrecht University, Utrecht, the Netherlands; 5 Health Psychology group, Institute of Applied Health Sciences, University of Aberdeen Aberdeen, Aberdeen, UK

**Keywords:** Condom use, Young people, Vignette study, Prevention, Sexually transmitted infections

## Abstract

**Background:**

Among young persons, declining condom use trends in many countries, high prevalences of chlamydia, and recent increases in gonorrhea diagnoses highlight the need to promote condom use. Individual social-cognitive factors and those of partners are known to influence condom use decision-making in steady partnerships, whereas research on casual partnerships is lacking. Our vignette study examined the influence of partner characteristics on condom use decision-making during casual vaginal sex.

**Methods:**

Dutch young people aged 16–24 (*n* = 1070) were recruited online in 2023. Participants indicated the likelihood of using a condom (‘If I were in this situation, I would use a condom’) after reading one of six randomly assigned partner vignettes. The last sentence of each vignette indicated the partner characteristics. The different partner characteristics in the vignettes were attitudes (positive/negative), norms (condom use/condom nonuse), and risk behavior (higher risk/lower risk). We performed three ANOVA analyses with Benjamini-Hochberg corrections; the dependent variable was the participant’s condom use decision, the three manipulated constructs were categorical between-participant independent variables. We explored interactions between the conditions and participant characteristics (i.e., demographic, social-cognitive and sexual behavior).

**Results:**

Participants were on average 19 years old, 55% was assigned female at birth. Participants were more likely to use a condom if the fictitious partner; had positive attitudes ηp²=0.17; indicated positive condom use norms ηp²=0.01; or engaged in more risk behavior ηp²=0.02. Different sexual behavior- and social-cognitive factors moderated these effects, e.g. condition effects were stronger in persons with more lifetime sexual partners or negative attitudes towards condom use. For example, participants who had more positive affective associations with condom use were less affected by a partner’s negative attitudes toward condom use.

**Conclusions:**

Findings demonstrate that new, casual partners can influence condom use decision-making in young people, especially the partner’s attitudes may be of importance. Our findings suggest that individuals go along with their partner’s attitudes or norms, and may be more inclined to use a condom based on higher risk behavior of the partner. Considering a partner’s influence and promoting positive attitudes towards condom use, specifically, but also promoting positive condom use norms may positively impact condom use.

**Supplementary Information:**

The online version contains supplementary material available at 10.1186/s12889-025-24741-6.

## Introduction

Condom use is a complex health behavior that can be influenced by multiple factors, among which social-cognitive factors likely play a significant role [1–4]. Though correct and consistent use of condoms can prevent sexually transmitted infections (STIs) [5], population-based data show that condoms are not always used [6–8]. Globally, declining trends in condom use among young people have been documented [9–13] and a recent WHO report highlights worrying decreases in condom use rates across Europe, Central Asia, and Canada. [14] For instance, in the Netherlands, only 53% of girls under 25 years old and 57% of boys reported using a condom during their most recent one-night stand in 2023, compared to 74% of girls and 85% of boys in 2012. [15, 16] As a result, more than a million STIs are estimated to be acquired globally each day [17], which has a major impact on global sexual and reproductive health. Of these STIs, chlamydia is the most commonly diagnosed bacterial STI worldwide [17], which, in the global North, is especially prevalent among heterosexually active adolescents and young adults[8, 18, 19]. In the Netherlands, population-based estimates of chlamydia prevalence indicate chlamydia being most prevalent in persons aged 18–19 (5.8%) and aged 20–25, (2.5%) compared with persons aged 25–35 (1.8%). [20] Given that chlamydia is linked to an increased risk of long-term reproductive health complications in women, [21–25], along with a recent rise in gonorrhea diagnoses among young heterosexuals in Europe [26] and an increase in gonorrhea positivity among tests in the Netherlands specifically, [27] there is a clear need for condom use promotion in this population.

What makes condom use different from many other health behaviors is that it occurs in a dyadic context. It is therefore not merely the decision of the individual, but rather a shared behavior that can be initiated by either one or both persons involved. While one partner may physically apply the condom, this decision can be shaped by both individuals. Accordingly, we aimed to assess how partner influences affect condom use decisions among young persons through a vignette study. In doing so, we aimed to gain more comprehensive insights into how different factors, notably sociodemographic and social-cognitive characteristics of both the individual and the partner jointly shape condom use. Specifically, we focused on the characteristics of the individual and their partner, including individuals’ views of condom use and their perceptions of their partner’s characteristics.

Prior research has identified sociodemographic differences in condom use. For example, women often indicate feeling less in control over condom use, possibly as a result of gender-based power dynamics or lower condom use self-efficacy. [28] Men tend to take more risks than women [29], which may stem from differences in beliefs about the consequences of the behavior. Next to gender-related factors, ethnicity, including cultural and religious factors, also plays a role in condom use. For example, certain machismo beliefs are associated with more condom use [30], while beliefs regarding, for example, the prohibition of premarital sex or condom use may influence condom use negatively [31]. This highlights how sociodemographic factors may impact condom use, underscoring the importance of considering such influences when studying condom use decision-making.

In addition to sociodemographic characteristics, individual social-cognitive factors are established correlates of condom use [1–3, 32]. The Theoretical Domains Framework (TDF) provides a validated theoretical framework grounded in 33 psychological theories (e.g. the Theory of Planned Behavior or the Health Belief Model), identifying key domains relevant to changing health behaviors. This framework facilitates a comprehensive approach to better understand the factors influencing behavior change across different situations and settings, [33–35] and has also been applied to sexual health[36, 37] The TDF includes, for example, social influences, emotion, and beliefs about capabilities, which relate to the key concepts of norms, attitudes, and self-efficacy that are central to the Reasoned Action Approach (RAA). [38] These attitudes, subjective norms and self-efficacy influence behavioral intentions, which in turn influence behavior [38, 39]. In the context of condom use, attitudes, subjective norms and self-efficacy have been established as strong correlates of condom use, emphasizing their importance. [3, 40–45]

The TDF also includes beliefs about consequences as a factor that can influence behavior. These beliefs refer to the perceptions of possible consequences of the behavior, for example, acquiring an STI. In condom use these beliefs are highly relevant, as for many individuals the sole purpose of using condoms is to prevent negative outcomes, such as STIs or unwanted pregnancies. Therefore, the perception of potential negative consequences can influence whether an individual will use a condom or not. A higher perceived risk of and susceptibility to STI/HIV are associated with higher levels of condom use, indicating that individuals who perceive a greater risk of negative outcomes are more likely to engage in protective behaviors such as condom use [1, 46], highlighting the role of beliefs about consequences in shaping condom use behavior.

Importantly, one’s perception of risk can be influenced by the type of sexual partner. Individuals’ thoughts and behavior regarding condom use often depend on whether the partner is a new, casual or regular partner. While condom use is important across all types of partnerships to e.g. protect against pregnancy or undiagnosed STIs, research has demonstrated higher condom use and condom use intentions with new/non-regular partners compared with regular partners [47–49]. This likely stems from a higher perceived risk of STI transmission from partners whose sexual history is less known. In a study among women it was indicated that these women felt more likely to contract an STI from a new partner, resulting in stronger condom use intentions [47]. Similarly, condom use attitudes tend to be more positive with non-primary partners than with primary partners [50], and condom use with a casual partner as sometimes regarded a social norm that one should follow [51]. Conversely, in steady relationships, condom use may even be seen as a sign of distrust [47, 51]. In addition, a shorter relationship duration has been associated with a higher likelihood of having disease prevention as a motive for condom use rather than pregnancy prevention. [52] These findings highlight differences in condom use based on the type of partner.

Though condoms are typically used in a sexual partnership between persons, research on determinants of condom use is generally focused on individuals. Consequently, there is limited understanding of how the social-cognitive characteristics of a partner influence the other partner and subsequent condom use, especially in non-steady or casual partnerships. Most existing research has examined individuals’ perceptions of their partner’s social-cognitive characteristics [1, 53–55]. For example, partner norms, as perceived by the individual, have been shown to predict condom use and condom use intentions in main relationships (i.e. a primary, steady partner). [53, 54] Similarly, perceptions of a main partner’s attitudes toward condom use strongly correlate with behavior, with negative partner attitudes being associated with lower rates of condom use. [56]

While these studies contribute to the understanding of the complexity of condom use in a dyadic context, most research focuses on established partners, despite casual sexual partners being of greater relevance from an STI transmission perspective. These differ from casual or new partners in aspects such as the level of commitment, trust, or communication. [43, 46, 53], In casual partnerships, these factors are often lower, necessitating different approaches to decision-making and negotiation around condom use. [47, 57].

This gap in the literature highlights a need for research that examines partner influences in casual partnerships. Although social-cognitive factors like norms and attitudes are known to influence condom use decisions, little is known about the impact social-cognitive characteristics of casual partners have on condom use. Addressing this gap is crucial for advancing our understanding of the nuances of condom use decision-making in higher-risk scenarios and for developing interventions tailored to these contexts.

In the present study, we build upon existing research investigating the influence of both individuals within one sexual dyad on condom use. Our study complements existing studies that have predominantly focused on established couples, [53, 54, 58] as we aim to gain more insights into condom use decision-making during vaginal sex in casual relationships. Due to ethical and practical impossibilities in this condom decision-making context, we employed experimental methodologies using a fictitious scenario, inspired by previous studies on condom use decision-making [59, 60]. Experimental methodologies have also been applied in the field of sexual discounting, which studies the devaluation of the benefits of safer sex, especially under conditions of delay or sexual arousal. Despite our study not focusing on delay or arousal, sexual discounting research is highly relevant to our context, as it demonstrates how partner characteristics, e.g. partner STI risk or desirability, can influence individuals’ willingness to use a condom.[61–63] However, this body of research primarily examines the process of discounting safer sex benefits, rather than the broader range of factors influencing condom use decision-making with casual partners. Furthermore, while vignette-based methodologies are commonly used in sexual discounting studies, these studies do not directly address how individuals make condom use decisions in casual sexual encounters outside the discounting framework. Our study directly addresses this gap, offering new insights into the factors that shape condom use decision-making beyond discounting effects.

In the current study, we used vignettes to systematically manipulate partner attitudes, social norms, and risk behavior and assess their influence on how likely the participants would be to use a condom. It should be clarified that our study aimed to examine condom use decision-making rather than intentions to use a condom. By presenting participants with realistic vignettes and asking them to make a choice about condom use in these specific scenarios, we aimed to capture the decision-making process itself, rather than simply measuring intentions. This focus allowed us to explore how various partner and individual factors may directly shape condom use decision-making in casual sexual partnerships. We have formulated the following hypotheses: Young people are more willing to use condoms if the partner has positive attitudes towards condom use (H1), if the partner expresses norms that are in favor of condom use (H2), or if the partner has engaged in more sexual risk behavior (H3). Since individual sociodemographic and social-cognitive variables may influence condom use decision-making, guided by the domains of the TDF and based on literature, we included relevant covariates (e.g. sexual behavior- or social-cognitive characteristics) that may interact with the effects of the vignette. With this study, we aim to gain more insights into how partner- and individual factors interact to influence condom use decision-making.

## Methods

### Procedure and participants

We conducted a between-subjects experimental study with two levels for each of the three separate independent variables (attitudes: positive/negative, norms: condom use/condom nonuse, risk behavior: high/low), resulting in six conditions. While the study independently manipulated attitudes, norms, and risk behavior to examine their effects, it does not represent a fully crossed factorial design (e.g., combinations such as positive attitudes with condom nonuse norms were not assessed). Instead, we compared key pairings within individual constructs (positive vs. negative attitudes, condom use vs. nonuse norms, and high vs. low-risk behavior) across the six scenarios. This structure allowed us to use the selected scenario combinations to evaluate the main effects of each independent variable on participants’ condom use decisions.

Participants were recruited online through convenience sampling, using a combination of paid advertisements on Instagram and Snapchat, through advertisements on www.sense.info (a Dutch website for young people regarding sexuality), and on TikTok by a Dutch nurse with a large young following, who answers questions regarding sexuality, puberty and related topics. These advertisements contained a link to the online survey, for which the platform Qualtrics was used. The survey language was Dutch level B1. Data were collected from the 24th of April 2023 until the 5th of June 2023. Participants were eligible if they were aged between 16 and 24 years old, and if they reported being heterosexual or bisexual. Participants not yet engaging in penile-vaginal sex were included, as the public health implications of this study should be applicable not only to those who are already sexually active, but also those who likely become sexually active in the near future. As penile-vaginal sex is not exclusive to cisgender hetero- or bisexual individuals, participants who identified differently (e.g. homosexual or non-binary) were eligible if they reported currently engaging in penile-vaginal sex or if they would likely do so in the future. No measures could be taken to ensure bots did not respond to the survey. After completion, participants could enter their email to participate in a raffle to win one out of 25 gift coupons worth 25 euros. The survey could only be filled in once per IP address to prevent multiple responses per person.

Throughout the survey, we did not explicitly state that we referred to external condoms. In the Netherlands, internal condoms are relatively unknown, largely unavailable in supermarkets and drugstores, and cost significantly more than external condoms, making them less likely to be used by young people. We therefore assumed participants only associated condom use with the external condom.

### Manipulations and measures

#### Vignettes

Using the Theoretical Domains Framework [33–35], we systematically identified predictors of condom use in the literature on correlates of condom use and on condom use interventions. We made a long list of factors that can influence condom use and then evaluated these predictors to determine their relevance and potential for modification within the vignettes. We focused on factors that could be effectively modified in a vignette to study their impact on condom use behavior. Taking these considerations into account, we included partner attitudes towards condom use (i.e., instrumental and affective attitudes) and partner norms regarding condom use (specifically descriptive norms, as these are more observable and realistic to manipulate than subjective norms) in the vignettes. In the third condition, we manipulated the partner’s previous relationships, with the intention of influencing participants’ risk perception. However, it is important to note that risk perception itself cannot be directly manipulated. Instead, we can only provide information or scenarios that may influence participants’ perceived risk. In line with theoretical frameworks, we assume that this perceived risk can influence condom use decision-making.

Participants were randomly assigned to one of the six vignettes. The vignette was adapted from a previous study on sexual risk decision-making [59] and slightly reworded (e.g. changing Frank/Rebecca to Fynn/Lynn) to align more closely with the current study and target population. In the vignette, participants run into an attractive acquaintance at a bar (either Lynn or Fynn – assigned based on the participant’s indicated sex as we focused on penile-vaginal sex) and after a fun evening, they go to the participant’s home at mutual consent. There is no mention of alcohol/substance use. After kissing for a while, the participant and Lynn/Fynn decide to have sex. The female in the vignette (either Lynn or the female participant) is on birth control. The vignette was equal for all 6 conditions apart from the last sentence. The last sentence read: ‘Lynn/Fynn says she/he finds it unimportant and annoying to use condoms’ (negative attitudes), ‘Lynn/Fynn says she/he finds it important and pleasant to use condoms’ (positive attitudes), ‘Lynn/Fynn says her/his friends use condoms during sex’ (condom use is the norm), ‘Lynn/Fynn says her/his friends don’t use condoms during sex’ (condom nonuse is the norm), ‘Lynn/Fynn had a long-term steady relationship before you’ (low risk behavior), or ‘Lynn/Fynn had multiple short-term relationships before you’ (high risk behavior). After this, in all vignettes, Lynn/Fynn asked what they should do. The full vignette can be found in the supplementary material.

#### Condom use likelihood

After reading the vignette, participants read “If I were in this situation, I would use a condom”. Answer options were presented on a 5-point scale (1 = no, 2 = probably not, 3 = neutral, 4 = probably yes, 5 = yes). There was no option to not have sex at all. We included a manipulation check, asking “Do you think Lynn/Fynn wants to use a condom?” (1 = no, 5 = yes). This check served to determine whether each of the manipulated conditions influenced the participant’s perception of the hypothetical partner’s condom use preference.

#### Sociodemographic characteristics

Demographical measures included age, sex assigned at birth (boy or girl), gender (boy, girl, between boy and girl, both boy and girl, neither boy nor girl, I don’t know (yet), or other, namely…) and sexual orientation (heterosexual, homo/gay, lesbian/gay, bisexual, pansexual, queer, asexual, I don’t know (yet), other, namely…). We made efforts to include sexually- and gender-diverse young people, as penile-vaginal sex is not limited to cisgender heterosexual persons. Participants who reported a gender different than their sex assigned at birth were asked whether they had either a penis or a vagina. Those with a penis were asked whether they (would) have sex with someone who has a vagina and vice-versa. Male participants who reported a sexual orientation other than heterosexual or bisexual were also asked whether they (would) have sex with someone who has a vagina, and female participants were asked if they (would) do so with someone who has a penis.

The level of education was indicated by the highest completed type of education according to the educational system of the Netherlands and categorized into lower, middle, and higher. Lower education level included primary education, prevocational secondary education, secondary vocational education level 1, and the first three years of senior general secondary education or pre-university secondary education. Middle education level included secondary vocational education levels 2, 3 or 4, and senior years of senior general secondary education or pre-university secondary education. Higher education level included higher vocational education and university. Furthermore, we assessed migration background (country/region of birth of participant and both their parents) and cultural background (country/region or culture of a country/region they felt most attached to; up to 2 countries could be selected).

#### Past sexual behavior

Data on sexual behavior included whether the participant ever had sex and age at sexual debut. Participants were also asked to report their number of lifetime sex partners and the number of sex partners in the past 12 months, including how many of those were new partners. Sex was defined as vaginal intercourse, which was repeated throughout the survey. Condom use in general was measured using a 5-point scale (1 = never, 5 = always). Participant’s current relationship status was not assessed in the study.

#### Social-cognitive characteristics

Social-cognitive measures that could potentially affect the partner’s influence on condom use decision-making in the vignettes were guided by the Theoretical Domains Framework (TDF) [35]. The rationale for using the TDF was to ensure a comprehensive yet theory-informed approach to explore several potential moderators of partner influences on condom use decision-making. While frameworks like the Theory of Planned Behavior or the Health Belief Model focus on a selection of behavioral predictors, the TDF synthesizes constructs from multiple behavioral theories, making the TDF suitable for our exploratory approach. We included determinants within the following domains: skills; memory, attention and decision processes; behavioral regulation; beliefs about capabilities; beliefs about consequences; intentions; goals; emotion; environmental context and resources; and social influences. The underlying theoretical constructs of the included TDF domains were selected based on commonly described correlates of condom use and on constructs that were targeted in condom use interventions that we previously studied in a systematic review. [64]Supplementary Table 1 presents the included domains with underlying (sub)constructs and the number of items and the corresponding questions. Certain questions were only displayed for participants who ever had sex. Items from validated measures were adapted to fit with our target behavior and context and changed into a 5-point scale. The questionnaire can be found in the supplementary materials.

### Data analysis

To examine whether participants in the conditions were similar in socio-demographic, social-cognitive- and sexual behavior characteristics including condom use, we used Kruskal-Wallis analyses. First, we performed an exploratory ANOVA analyses including all six conditions. The main results were assessed using three ANOVA analyses, and Benjamini-Hochberg corrections were applied to the P-values. In all three analyses, the dependent variable was the participant’s condom use decision, and the three manipulated constructs (attitudes, norms, and risk behavior) were categorical between-participant independent variables. For the manipulation checks, we used three ANOVA analyses, we checked whether the 2 levels for partner attitudes, partner norms and partner risk behavior, respectively, affected perceived partner preferences. The dependent variable was the participant’s response to whether they thought Lynn/Fynn wants to use a condom.

In additional separate analyses, the above-described individual demographical-, social-cognitive- and sexual behavior measures were added to the univariate ANOVA analyses to explore possible interactions. Demographical- and sexual behavior measures were selected based on literature, focusing on factors that have been associated with condom use. Social-cognitive factors were guided by the TDF, focusing on constructs that have been shown to correlate with condom use. Table 1 provides an overview of the demographical- and sexual behavior measure that were included as covariates, Table 2 displays the social-cognitive factors included as covariates.

**Table 1:** Sociodemographic characteristics and sexual- and STI-testing behavior among dutch study participants, 2023

	Females		Males		Total	
	n	%	n	%	N	%
Age						
16–18	266	45	227	48	493	46
19–21	225	38	216	45	441	41
22–24	102	17	33	7	135	13
Gender						
Cisgender	571	96	465	98	1036	97
Gender-diverse	22	4	9	2	31	3
Sexual orientation						
Heterosexual	436	74	430	91	866	81
Not heterosexual	155	26	45	9	200	19
Education						
Low	258	44	220	46	478	45
Middle	230	39	211	44	441	41
High	105	18	46	10	151	14
Migration background						
No migration background	442	75	365	77	807	75
Migrant	53	9	31	7	84	8
Child of migrant(s)	98	17	80	17	178	17
Cultural background						
Dutch only/primarily	539	91	445	93	984	92
Dutch secondarily/non-Dutch	54	9	31	7	85	8
Ever had sex						
Yes	465	78	341	71	806	75
No	126	21	133	28	259	24
Age of sexual debut*						
13–15	148	32	133	43	281	36
16–18	257	55	148	48	405	52
19–24	61	13	27	9	88	11
Condom use in general*						
Always	103	22	68	20	171	21
Most of the time	81	17	67	19	148	18
Sometimes	55	12	54	16	109	13
Usually not	126	27	76	22	202	25
Never	102	22	79	23	181	22
Condom use in general, among people with new partners						
Always	77	0	45	0	122	0
Most of the time	64	0	43	0	107	0
Sometimes	45	0	43	0	88	0
Usually not	82	0	82	0	164	0
Never	59	0	59	0	118	0
Number of lifetime sexual partners*						
1–2	263	56	172	50	435	54
3–5	102	22	94	27	196	24
6–9	42	9	32	9	74	9
10–14	32	7	22	6	54	7
15+	28	6	24	7	52	6
Number of recent sexual partners**						
0–1	298	67	180	58	478	63
2–3	89	20	88	29	177	23
4+	61	14	40	13	101	13
total	448		308		756	
STI test and result*						
Not tested	309	79	279	66	588	74
Tested***	155	21	61	33	216	26
Negative	111	73	48	79	159	74
Chlamydia	30	20	8	13	38	18
Other STI	1	1	3	5	4	2
Chlamydia and other STI***	10	3	1	1	11	3

* Among individuals reporting ever having had sex.

** Among individuals reporting recent (past 12 months) sex.

*** Numbers of individuals with STI test results do not add up to the total of individuals tested for STI. STI result percentages are among individuals who reported their STI test result.

**Table 2:** Descriptives of social-cognitive characteristics among Dutch young people (16–24) in 2023.*

Variable	Sex	Mean	SD	Median	Q1	Q3	Minimun value	Maximum value
Self-confidence	Combined Sexes	3.58	0.97	4	3	4	1	5
	Females	3.40	0.93	4	3	4	1	5
	Males	3.80	0.97	4	3	4	1	5
Self-efficacy	Combined Sexes	4.22	0.90	4	4	5	1	5
	Females	4.11	0.92	4	4	5	1	5
	Males	4.35	0.86	5	4	5	1	5
Negotiation self-efficacy	Combined Sexes	4.22	0.88	4	4	5	1	5
	Females	4.20	0.87	4	4	5	1	5
	Males	4.24	0.89	4	4	5	1	5
Intentions	Combined Sexes	3.54	1.27	4	3	5	1	5
	Females	3.61	1.24	4	3	5	1	5
	Males	3.44	1.31	4	3	5	1	5
Response efficacy	Combined Sexes	11.73	2.37	12	10	13	3	15
	Females	11.61	2.26	12	10	13	3	15
	Males	11.87	2.49	12	11	14	3	15
Perceived severity	Combined Sexes	4.33	0.97	5	4	5	1	5
	Females	4.39	0.95	5	4	5	1	5
	Males	4.25	0.98	5	4	5	1	5
Normative influences								
Injunctive normative influences	Combined Sexes	3.83	1.07	4	3	5	1	5
	Females	3.93	1.01	4	3	5	1	5
	Males	3.71	1.12	4	3	5	1	5
Descriptive normative influences	Combined Sexes	3.26	1.01	3	3	4	1	5
	Females	3.21	1.01	3	3	4	1	5
	Males	3.32	1.02	3	3	4	1	5
Habitual behavior	Combined Sexes	10.37	4.82	10	7	14	4	20
	Females	10.56	4.77	10	7	14	4	20
	Males	10.11	4.88	9	6	14	4	20
Memory in the heat of the moment	Combined Sexes	2.48	1.30	2	1	3	1	5
	Females	2.44	1.26	2	1	3	1	5
	Males	2.53	1.35	2	1	4	1	5
Memory during intoxication	Combined Sexes	2.57	1.37	2	1	4	1	5
	Females	2.48	1.31	2	1	3	1	5
	Males	2.69	1.43	3	1	4	1	5
Associations (attitudes)								
Overall	Combined Sexes	4.19	1.05	5	3	5	1	5
	Females	4.29	0.98	5	4	5	1	5
	Males	4.05	1.11	5	3	5	1	5
Affective	Combined Sexes	5.31	2.12	5	4	6	2	10
	Females	5.57	2.11	6	4	6	2	10
	Males	4.98	2.09	5	4	6	2	10
Instrumental	Combined Sexes	8.65	1.54	9	8	10	2	10
	Females	8.77	1.45	9	8	10	2	10
	Males	8.50	1.63	9	8	10	2	10

* For all variables, higher values indicate more favorable outcomes.

To explore whether demographic, sexual behavior, and social-cognitive covariates moderated the effect of condition on condom use decision-making, we conducted separate interaction analyses for each covariate within each of the three manipulated constructs (attitudes, norms or risk behavior). To control for multiple testing, we applied the Benjamini-Hochberg correction instead of Bonferroni. The Benjamini-Hochberg procedure controls the false discovery rate, which is suitable for exploratory analyses where multiple hypotheses are tested, since it reduces the likelihood of identifying false positives while maintaining sensitivity to detect potential effects. Only Benjamini-Hochberg-adjusted P-values were reported.

 Significant interaction effects were further analyzed using comparisons of values 1 standard deviation (SD) above and below the mean (M)[65]. Statistical significance for all analyses was set at p < 0.05. All analyses were performed using R. Post-hoc power analyses for the main ANOVA analyses were performed using the ‘pwr’ package.

### Ethics approval and consent to participate

The Netherlands National Medical Ethics Committee, NedMec, reviewed our study and found it to not be subject to the Medical Research Involving Human Subjects Act (WMO), meaning that the study did not need further reviewing by the Medical Ethics Committee #22/760). Our study was approved by the Human Research Ethics Review Board of the Faculty of Social & Behavioural Sciences, Utrecht University (#22-0439), guided by the National Code of Ethics for Research in the Social and Behavioural Sciences involving human participants, as well as the Netherlands Code of Conduct for Research Integrity and the ethical principles outlined in the Declaration of Helsinki. All participants provided informed consent, no IP-addresses were collected, and their privacy and data were handled in compliance with the General Data Protection Regulation. At the start of the survey, participants received all information required to be able to provide informed consent. Participants who did not provide informed consent did not receive further questions are were excluded from the survey. As per Dutch law, young people aged 16 or 17 did not need parental consent.

## Results

### Participant characteristics

In total, 1,995 participants, of whom 1,070 met the inclusion criteria and completed the entire questionnaire. Participants were on average 19 years old (SD = 2.1) and a little over half were assigned female at birth (55%), identified as cisgender (97%), had a lower (44%) or middle (39%) level of education, had no migration background (75%), and had a Dutch cultural background (81%). Since none of the gender-diverse participants reported having had surgery to change their genitalia, we reported results stratified by the sex assigned at birth. Most participants had ever engaged in penile-vaginal sex (75%), had a total number of lifetime sexual partners of 1-2 (54%), and never or usually did not use condoms (47%). The mean age of sexual debut was 16 years old (Table 1).

Across conditions, participants were similar in demographical characteristics and most social-cognitive- and sexual behavior variables. Conditions were randomly allocated, each group containing approximately one sixth of the study sample; precise proportions ranged from 16.2% to 17.5% (n= 173-187 participants per group).Groups differed in the proportion of participants who reported ever having had sex χ2 = 11.60, p <. 05, relatively more participants in the positive attitudes condition ever had sex (82%) and relatively fewer participants in the low-risk behavior condition ever had sex (68%). The proportion of those reporting having had sex ranged between 73% and 80% for the other conditions. The value for instrumental associations also slightly differed between groups χ2 = 14.40, p <. 05. The mean value of instrumental associations, 8.3, was slightly lower in participants in the high risk condition compared with the other conditions where the mean ranged between 8.5 and 8.9. Table 2 presents descriptives characteristics of social-cognitive factors.

### Perceived partner condom preference

Regarding the manipulation checks, as expected, within the attitudes conditions, participants who read the vignette with positive attitudes were significantly more likely to expect the partner to want to use a condom F(1,347) = 906.6, p <.001, ηp²= 0.72. Similarly, participants who read the vignette in which condom use was the norm also reported significantly higher values of their expectations on whether the partner wanted to use a condom. F(1,367)= 233.8, p <.001, ηp²= 0.39. For the risk behavior vignette, there was no significant effect of the condition on the expected partner condom use preference F[1,350] = 0,79, p = 0.37, ηp²= 0.002.

### Effect of partner characteristics on condom use likelihood

**Fig. 1:**
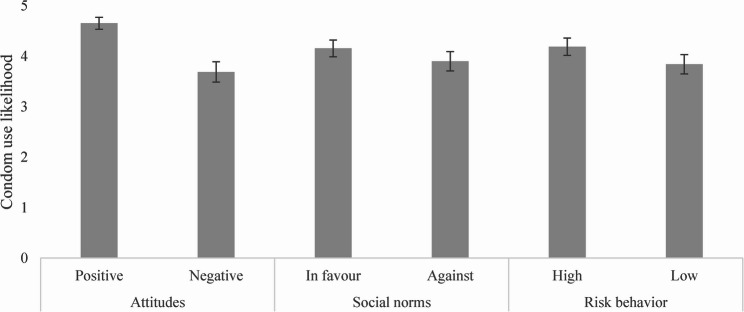
Mean and 95% confidence intervals of condom use likelihood per condition

The overall mean value of condom use likelihood was 4.07 (SD = 1.21). Figure 1 displays the mean values and 95% confidence intervals for each condition. The ANOVA analysis including all six conditions demonstrated significant differences in condom use likelihood; F(5, 1064) = 15.1, p < 0.001, ηp² = 0.07 (Table 3). As expected, within the three constructs we found significant differences in condom use likelihood; participants were more likely to use condoms when the partner had positive attitudes towards condom use (Hypothesis 1) F(1, 347) = 69.01, p < 0.001, ηp² = 0.17, when condom use was the partner’s norm (Hypothesis 2) F(1, 367) = 4.04, p = 0.045, ηp² = 0.01 or when the partner engaged in more risk behavior (Hypothesis 3) F(1, 350) = 7.34, p =0.007, ηp² = 0.02 (Table 3). The attitudes conditions yielded a large effect size, [66] whereas the norms and risk behavior conditions yielded small effect sizes. Post-hoc power analyses yielded a power of 1 for the ANOVA assessing all six conditions, 1 for the attitudes conditions, 0.51 for the norms conditions and.78 for the risk behavior conditions.

**Table 3:** Main ANOVA analyses results of the analysis including all six conditions and the three vignette-specific analyses

		M	SD	Df		Partial eta square	F-value	*P*-value
				Condition	Residuals			
All conditions		-	-	5	1064	0.07	15.1	**< 0.001**
		-	-					
Attitudes	Positive	4.65	0.79	1	347	0.17	69.01	**< 0.001**
	Negative	3.69	1.32					
Norms	In favour	4.16	1.13	1	367	0.01	4.04	**0.045**
	Against	3.90	1.30					
Risk behavior	Higher	4.19	1.13	1	350	0.02	7.34	**0.007**
	Lower	3.84	1.27					

All p-values presented have been corrected using the Bejanimi-Hochberg procedure

*M*  Mean, *SD* Standard Deviation, *Df*  Degrees of freedom

### Sensitivity analyses

Participants in the different conditions only differed significantly on the variable of ever having had sex, which we assumed to be based on chance. Sensitivity analyses including ever having had sex as a covariate yielded similar results.

### Moderators of effects on condom use likelihood

For the attitudes vignettes, eight variables were found to have a significant moderating effect (Table 4). Only significant effects are reported in the figures and tables. The simple slopes of these moderating effects are demonstrated in Supplementary Figures S1-S8, which indicate that the effect of the attitudes vignette on condom use likelihood was stronger (i.e., a lower condom use likelihood for the negative attitudes condition and a higher condom use likelihood for the positive attitudes condition) in participants who had: lower prior condom use intentions (Figure S1); a lower ability to remember to use a condom in general (memory, Figure S2) or during intoxication (memory during intoxication (Figure S3); indicated that condom use was less of an habitual behavior (Figure S4); or less positive attitudes towards condom use (Figure S5). For the number of lifetime- or recent partners, condom likelihood was similar across different number of partners in the positive attitudes vignette, but a lower condom likelihood was observed as the number of partners increased (Figures S6&S7). For response efficacy, the condom use likelihood was similar across different levels of response efficacy for the negative attitudes condition, but the condom use likelihood was higher as response efficacy became greater (Figure S8).

**Table 4:** Results of univariate ANOVA analyses per interaction with the partner attitudes condition

		Df	F-value	Partial eta square	P-value
Number of lifetime partners	Condition	1	51.47	.16	<0.001
	Number of lifetime partners	1	14.13	.05	<0.001
	Condition * number of lifetime partners	1	4.19	.02	0.042
	Residuals	249			
Number of recent partners	Condition	1	48.57	.15	<0.001
	Number of recent partners	1	19.88	.08	<0.001
	Condition * number of recent partners	1	6.56	.03	0.011
	Residuals	223			
Condom use intentions	Condition	1	76.05	.18	<0.001
	Condom use intentions	1	31.91	.08	<0.001
	Condition * condom use intentions	1	5.49	.02	0.020
	Residuals	345			
Response efficacy	Condition	1	70.41	.17	<0.001
	Response efficacy	1	4.39	.01	0.037
	Condition * response efficacy	1	4.67	.01	0.037
	Residuals	345			
Habitual behavior	Condition	1	53.25	.18	<0.001
	Habitual behavior	1	21.41	.08	<0.001
	Condition * habitual behavior	1	6.17	.02	0.014
	Residuals	249			
Memory	Condition	1	52.82	.18	<0.001
	Memory	1	19.57	.07	<0.001
	Condition * memory	1	5.76	.02	0.017
	Residuals	249			
Memory during intoxication	Condition	1	53.11	.18	<0.001
	Memory during intoxication	1	16.75	.06	<0.001
	Condition * memory during intoxication	1	10.11	.04	0.002
	Residuals	249			
Affective associations	Condition	1	75.59	.18	<0.001
	Affective associations	1	29.56	.08	<0.001
	Condition * affective associations	1	5.56	.02	0.019
	Residuals	345			

All p-values presented have been corrected using the Bejanimi-Hochberg procedure. Only significant moderation effects are presented

*M* Mean, *SD* Standard Deviation *Df * Degrees of freedom

For the norms vignettes, three variables demonstrated significant interactions (Table 5). The effect of the norms condition was stronger in participants with a higher number of lifetime partners (Figure S9). The condom use likelihood was minimally affected by the norms conditions in participants with more favorable descriptive norms or more positive attitudes (Figures S10&S11), whereas those with less favorable norms and negative attitudes reported lower condom use likelihood in the condom nonuse norms condition.

**Table 5:** Results of univariate ANOVA analyses per interaction with the partner norms condition

		Df	F-value	Partial eta square	P-value
Number of lifetime partners	Condition	1	2.10	.01	0.150
	Number of lifetime partners	1	3.81	.01	0.078
	Condition * number of lifetime partners	1	7.19	.02	0.023
	Residuals	281			
Descriptive normative influences	Condition	1	4.15	.01	0.042
	Descriptive normative influences	1	8.05	.02	0.014
	Condition * descriptive normative influences	1	4.23	.01	0.042
	Residuals	365			
Affective associations	Condition	1	4.72	.01	0.030
	Affective associations	1	58.52	.14	<0.001
	Condition * affective associations	1	5.45	.01	0.030
	Residuals	365			

All p-values presented have been corrected using the Bejanimi-Hochberg procedure. Only significant moderation effects are presented

*M* Mean, *SD* Standard Deviation, *Df*  Degrees of freedom

 For the risk behavior vignettes, three variables were found to significantly interact with the conditions (Table 6). The condom use likelihood was less affected by the risk behavior conditions in participants with a better ability to remember to use a condom in general (memory, Figure S12) or with more positive attitudes (Figures S13). The condition effect increased as the number of lifetime partners increased; across different numbers of partners, differences in the condom use likelihood for the high risk condition were relatively small (Figure S14). For the low risk condition, however, these differences were larger and participants with more partners reported lower condom use likelihood. None of the demographic variables were found to have a moderating effect.

**Table 6:** Results of univariate ANOVA analyses per interaction with the partner risk behavior condition

		Df	F-value	Partial eta square	P-value
Number of lifetime partners	Condition	1	5.49	.03	0.020
	Number of lifetime partners	1	21.30	.07	<0.001
	Condition * number of lifetime partners	1	6.46	.02	0.017
	Residuals	269			
Memory	Condition	1	5.58	.03	<0.001
	Memory	1	28.40	.10	0.028
	Condition * memory	1	3.94	.01	0.048
	Residuals	269			
Attitudes combined	Condition	1	10.20	0.06	0.002
	Attitudes combined	1	134.25	0.28	<0.001
	Condition * attitudes combined	1	4.09	0.01	0.044
	Residuals	348			

All p-values presented have been corrected using the Bejanimi-Hochberg procedure. Only significant moderation effects are presented

*M* Mean, *SD* Standard Deviation, *Df* Degrees of freedom

## Discussion

In this study, we examined partner influences on condom use decision-making in six different partner vignettes. The partner’s attitudes, norms, and sexual risk behavior all affected condom use likelihood; the condom likelihood was higher with positive attitudes towards condom use, condom use as the norm or high-risk behavior. Moderation analyses demonstrated that for each of the three constructs, different social-cognitive- and sexual behavior factors of the individual affected the influence the partner had on condom use decision-making. The number of lifetime partners and individual attitudes towards condom use showed an interaction effect with all three constructs.

 Our findings confirm the influence of a partner on condom decision-making. This builds on previous research [53, 54, 58] on partner influences on condom use in established couples, indicating its persistence even within novel, casual partnerships. The influence of either the partner’s attitudes or norms on condom decision-making demonstrated that also in a hypothetical situation with a casual and relatively unknown partner, individual condom use decision-making is influenced by a partner’s social-cognitive characteristics. The Reasoned Action Approach (RAA) describes how attitudes towards a behavior are influenced by behavioral beliefs and the evaluation of behavioral outcomes. A partner indicating positive attitudes towards condom use may influence one’s beliefs and may reinforce the perceived benefits of condom use. Also, if the partner indicated to find it pleasant to use condoms, the participant may have expected to experience more sexual pleasure when using condoms. These findings support the RAA, as they suggest that a partner’s beliefs may influence the individual’s beliefs, in turn influencing condom use. On the other hand, if the partner indicated to find condoms unpleasant, individuals may expect more sexual pleasure by not using a condom. Research has demonstrated condom nonuse to be associated with an expected reduction of sexual pleasure and the perception that a partner disapproves of condom use. [67] Furthermore, a study among adolescent women has found that women who felt condoms interfered with either their own or their partner’s pleasure had lower condom use self-efficacy and lower self-efficacy to refuse condomless sex. [68] This may also be true when considering the partner’s norms; individuals may expect a more pleasant outcome when complying with the norms their partner has, which aligns with the RAA construct of evaluation behavioral outcomes. The partner’s norm may also have influenced individual norms through normative beliefs and motivation to comply. The finding that the sexual risk behavior of a partner also influenced condom use decision-making may also be explained by its influence on the evaluation of behavior outcomes that can influence individual attitudes. Our findings suggest that individuals feel a need to protect themselves for STIs when the partner has potentially engaged in more risk behavior, which is in line with previous research [46, 47]. To conclude, our findings demonstrate an influence of a partners attitudes, norms or risk behavior on condom use decision-making, possibly through influencing individual attitudes and norms.

 The difference in condom use likelihood was the largest between the two attitude vignettes. The partner’s attitudes demonstrated stronger effects (large effect size) on condom use likelihood than the partner’s norms (small effect size), though we did not statistically test for differences between the attitudes and norms conditions. In the vignettes, the partner’s attitudes reflected the partner’s feelings and thoughts on condom use, whereas the partner’s norms reflected the behavior of the partner’s friends. Attitudes may thus have been a more direct representation of an individual's thoughts and feelings than norms, which may imply but not directly reflect the partner’s cognitions. Previous research consistently showed that individual attitudes have a stronger influence on condom use intentions than social norms [3, 69, 70]. This may also be true for the attitudes and norms of a partner. It is important to distinguish between the different manipulations, as the large effect size associated with partner attitudes indicate a potentially important role in shaping condom use decisions. Consequently, preventative efforts may benefit most from improving attitudes towards condom use.

 We also found that the attitudes of the partner had stronger effects on condom use likelihood than the perceived sexual risk behavior of the partner, as manipulating the partner attitudes resulted in a large effect size while the risk behavior manipulation resulted in a small effect size. The sexual risk behavior vignettes did not provide any information on prior condom use of the partner and therefore the person’s risk perception relied solely on the number and duration of relationships. In the attitudes vignette, however, the partner explicitly mentioned their preference. On the one hand, this may explain the difference in the impact the vignettes had on condom use decision-making. On the other hand, it is more realistic that one has knowledge solely on the relationship history of their partner than on both their relationship and condom use history. It can also be hypothesized that the thoughts and feelings of the partner on using a condom may be more influential in condom use decision-making than a partner’s sexual behavior and the individual’s corresponding perceived risk of contracting an STI. In the vignettes, there was no risk for pregnancy and therefore the main reason to use a condom would be to prevent STIs. This suggests that complying with a partner’s preference may be perceived as more important than protecting oneself against possible STIs. It emphasizes the need to not solely focus on STI risk in preventive interventions but to also take into account the partner’s preferences and the surrounding communication.

Our study found multiple different social-cognitive- and sexual behavior characteristics to influence the effect the vignette had on condom use decision-making. The number of lifetime partners of the individual was found to interact with condom decision-making for all vignettes, with the effects of the vignette being stronger in persons with more lifetime partners. This may seem counterintuitive, as it suggests that those who have made more condom use decisions with new partners are more easily influenced by their partner.

However, having more experience with condom use decisions may have enabled the person to make a more realistic judgment on their condom use in that specific context. Indeed, research on decision-making across different topics has demonstrated that individuals with more experience are better able to make judgments in a hypothetical situation. Notably, this effect did not persist for the number of recent partners, which supports the idea that it is the cumulative rather than solely recent experience of making condom decisions that enables individuals to better imagine and evaluate their own likely behavior in that situation. Efforts to further understand and promote condom use may therefore benefit from taking into account the level of familiarity with condom decision-making.

We also found that the individual’s own attitudes towards condom use affected condom use decision-making in all three conditions. If the partner’s attitudes or norms were not in favor of condom use or if the partner engaged in less risky sexual behavior, the likelihood of using a condom was lower when the participant’s own attitudes were not in favor of condom use than if the individual held more positive attitudes towards condom use. This implies that individual attitudes towards condom use could either strengthen or weaken the influence a partner has on condom use decision-making. In other words, findings imply that individuals with less positive attitudes are more susceptible to a partner’s influence, and are especially more likely to not use condoms when the partner has negative attitudes towards condom use. A similar effect of the individual’s condom use intentions was seen: the effect of the attitudes manipulation was stronger in individuals who had lower condom use intentions. Individuals who had greater condom use intentions were less likely to be negatively impacted by the negative attitudes of the partner. These findings have important implications for condom promotion interventions. Though it is most likely that a condom will be used when both partners have attitudes or norms that are in favor of condom use, improving the attitudes of just one individual in the partnership may already positively impact condom use. Also, the lesser influence of the partner on condom use likelihood in individuals with greater condom use intentions implies that individuals intentions may be more resistant to negative influences of a partner, thus promoting condom use intentions may also impact condom use. Future research should further investigate the magnitude of this impact.

Several strengths and limitations of our study can be identified. A strength of this study is its design using vignettes, which enabled us to systematically manipulate the characteristics of the partner. Since it would be unethical to conduct a similar study in a real-life setting, the use of vignettes can provide an approximation of what may happen in real life. In addition, we were able to recruit a large and diverse study sample, which enhances the generalizability of our findings. Though there will likely always be a bias in who agrees to participate in (sex-related) research, it is worth noting that our study succeeded in including a sample that is demographically representative of the Dutch population in terms of migration background [71], and sexual orientation and gender identity [15]. In a population-based study of young people under the age 25 in the Netherlands, 26% of young people reported to always have used a condom with the last partner [15], in our study we found that 21% reported to always use a condom in general. Note that the population-based study assessed condom use with the most recent partner, which may reflect a particular context or situation, whereas our study assessed condom use in general, which may capture a broader or more habitual behavior. As the population-based study also included 13-15-years olds (the age group with the highest level of condom use), condom use among 16-24-years olds would likely be lower than 26%. Condom use in our sample appears to be comparable to findings from this national study, although direct comparisons should be interpreted with caution due to differences in how condom use was measured.

A limitation of our study is its hypothetical character. In the vignettes, certain information about the partner was provided, which may not be completely realistic life. In a real-world setting, information on the partner is likely limited. However, it does also highlight the importance of communication in condom decision-making: one cannot be made aware of their partner’s preferences when the partner does not communicate these. This may be especially relevant to share one’s attitudes, as the partner’s attitudes had the largest influence on condom decision-making and sharing one’s own attitudes may be more realistic than sharing one’s perceived norms or previous risk behavior. Another limitation of our study is that we studied the individual’s expected condom decision in a certain scenario, which may be somewhat comparable to behavioral intentions, but is conceptually distinct. While intentions refer to one’s plan to perform a behavior in the future, a decision in a specific context involves making a choice when presented with concrete circumstances and options. Although intentions to use condoms are a strong predictor of actual condom use behavior [32, 72], the well-known intention-behavior gap [73] underscores that there can be a discrepancy between intentions and actual behavior. Specifically, while intentions are predictive, other factors, e.g. past behavior/habit [74, 75], can interfere with translating intentions into action. Research has described this gap being most existent among intenders, as they do not always end up performing the intended behavior, whilst non-intenders are likely to not perform the behavior [76]. Our study’s focus on decision-making in specific contexts may more closely approximate real-life choice than intentions, but it remains a hypothetical measure that may not fully reflect actual behavior. This highlights the limitation that our study relies on presumed behaviors, which may not always translate into actual behavior. Also, a valid manipulation check for the risk behavior manipulation was missing, thus limiting our ability  to determine whether this manipulation was successful. Consequently, the interpretability of findings regarding the influence of partner risk behavior is constrained, and future research should include a manipulation check to specifically address STI risk to clarify this issue. Also, post-hoc power analyses revealed sufficient to excellent power for most analyses. For the norms conditions, power was relatively low. Although a significant effect was found, the low statistical power suggests that the observed effect size should be interpreted with caution. Future research with larger samples is needed to confirm the robustness and magnitude of this effect. Lastly, since we aimed to explore possible interaction effects, we conducted a high number of statistical tests. Although this increases the risk of false positive associations arising by chance, we expect this risk to be minimized as we applied Benjamini-Hochberg corrections. Another limitation is that we were not able to look at differences between participants based on their current relationship status, as this information was not collected. The scenario asked participants to imagine themselves being single and having a casual sexual contact, it is possible that those currently in relationships may have found it more difficult to fully engage with this hypothetical situation. This may have influenced the effectiveness of the manipulations and consequently participants’ responses. Future research should consider assessing and controlling for current relationship status to establish whether this interferes with the manipulation.

Recommendations for future research include conducting studies that vary the vignette setting. For example, in our vignette, the encounter took place at a bar. We did not mention the use of alcohol or other substances, though research has demonstrated that this affects condom use decision-making [77]. Since nightlife is a common setting to meet new sexual partners, future studies considering both sober and intoxicated scenarios could provide relevant insights. Not only sober and intoxicated states would be interesting, but also different settings (e.g., at a house party or on a campsite) and the extent to which the partner  is familiar (a well-known friend or someone they just met). This would increase the generalizability of findings and provide insights in which preventive efforts may be beneficial in specific contexts. Also, a mixed between- and within-subjects factorial design including different combinations of the partner characteristics in the vignettes, as frequently employed in sexual discounting studies,[61] could provide additional insights into how these characteristics interact in shaping condom use decision-making. While in hindsight this factorial approach might have been possible, we anticipated a much lower response rate and therefore determined that we would have insufficient power.

Implications of our study are of special interest when aiming to promote condom use among a young population. Our findings demonstrate that hypothesized condom use can be increased when a sexual partner has attitudes that are in favor of condom use or when condom use is the partner’s norm. This implies that attitudes and norms are possible targets in condom promotion. Especially attitudes may be a suitable target, as the strongest effects of the partner on condom use decision were seen in the attitudes vignettes. In addition, the individual’s own attitudes towards condom use significantly influenced condom use decision-making. This implies that one’s own attitudes could either further negatively impact condom use when the partner is against condom use, or weaken a partner’s negative influence on condom use when the individual holds positive attitudes towards condom use. However, in the vignettes, the partner explicitly communicated either their attitudes or the norms they perceive. In reality, there may not always be a conversation prior to sexual intercourse about condom use, as research shows young people often find it difficult to discuss or negotiate condom use [78

Similarly, though our findings showed persons were more likely to intend to use a condom when the hypothetical partner engaged in more sexual risk behavior, in reality, an individual may not be aware of a partner’s prior sexual behavior. This could also be discussed prior to sexual intercourse, however, this may be even more difficult to bring up. Our findings suggest that participants likely assumed a partner with one long-term partner to pose less STI risk than a partner with multiple short-term partners. However, these assumptions may not always accurately reflect STI risk, and can be problematic when they lead to reduced condom use. It therefore remains important for public health interventions to not solely focus on addressing gaps in STI knowledge, but to also promote accurate risk perceptions.

## Conclusion

 In conclusion, using vignettes on a sexual encounter with a new partner, we were able to systematically vary the partner’s attitudes, norms, and risk behavior and determine how this impacts condom decision-making. Our findings confirmed that, not only in established couples but also in new partners, the partner has a significant influence on condom use decision-making, especially the attitudes of the partner may have a profound impact on shaping condom use. These insights have several implications. Sexual health professionals and counselors may use these findings to address communication between partners and the influence of partner attitudes and norms, and to discuss misconceptions about STI risk based on relationship history. Efforts to improve sexual health should consider the partner’s influence on condom use. In addition, policy efforts such as sexual education curricula, could require the inclusion of communication- and negotiation skills to equip individuals with tools to navigate these interactions. Public health initiatives and sexual education should not only focus on providing basic information about condom use and STI prevention, but also highlight the importance of communication regarding condom use and understanding how a partner’s perspective can shape condom use behavior. Specifically, by promoting and communicating positive attitudes towards condom use, establishing condom use as the norm, and addressing partnership dynamics related to condom use, interventions may positively impact condom use behavior and improve sexual health outcomes.

## Supplementary Information

Supplementary Material 1.

## Data Availability

Data are available upon reasonable request.

## Abbreviations

STIs: Sexually transmitted infections
TDF: Theoretical Domains Framework
RAA: Reasoned Action Approach
TPB: Theory of Planned Behavior
