# Supplementary material for: Investigating the influence of partner attitudes, norms and risk behavior on condom use decision-making during penile-vaginal sex with casual partners: a vignette study among dutch young people
Source: BMC Public Health. 2025 Oct 8;25:3388. doi: 10.1186/s12889-025-24741-6 (PMC12505808; doi:10.1186/s12889-025-24741-6)

Supplementary text S1, vignettes, translated from Dutch

Scenario for female:

You are single and you encounter an attractive acquaintance, Fynn, in a bar. You chat for a while and both notice that it's a pleasant conversation. Fynn is funny and genuinely interested in what you have to say. You spend the whole evening together. At the end, he walks you home. You ask if he wants to come in, and he says yes. After chatting and kissing on the couch for a while, it's clear that you both want to have sex with each other. [sentence that varies for each condition]

Scenario for male:

You are single and you encounter an attractive acquaintance, Lynn, in a bar. You chat for a while and both notice that it's a pleasant conversation. Lynn is funny and genuinely interested in what you have to say. You spend the whole evening together. At the end, she walks you home. You ask if she wants to come in, and she says yes. After chatting and kissing on the couch for a while, it's clear that you both want to have sex with each other. [sentence that varies for each condition]

Conditions:

- Attitudes:
  - Positive: Fynn/Lynn says that he/she finds it important and pleasant to use condoms and asks, "what shall we do?"
  - Negative: Fynn/Lynn says that he/she finds it unimportant and unpleasant to use condoms and asks, "what shall we do?"
- Norms:
  - Positive: Fynn/Lynn says that his/her friends have sex with condoms and asks, "what shall we do?
  - Negative: Fynn/Lynn says that his/her friends have sex without condoms and asks, "what shall we do?"
- Risk behavior:
  - Low: Fynn/Lynn has only had one long-term relationship before you and asks, "what shall we do?"
  - High: Fynn/Lynn has had multiple short-term relationships before you and asks, "what shall we do?"

Supplementary text S2, questionnaire, translated from Dutch

**Thank you for participating in this study!**

**What is this questionnaire about?**

This questionnaire is about sex, sexual behavior, and the use of condoms. We are asking you and many other young people to answer these questions.

**Who can participate?**

Young people in the Netherlands aged 16 to 24 years old.

It doesn’t matter whether you have had sex or not.

**Why are we asking these questions?**

We want to know how young people think about condoms and when they do or don’t use them. This way, we can provide better information to young people or help them with their problems.

**Do you have to participate?**

Participation in this study is voluntary. You can stop participating at any time if you no longer wish to continue.

**What happens to your answers?**

Your information will be treated confidentially. The researchers cannot see who you are.

**How do you participate?**

- Answer the questions and use the arrows at the bottom to go to the next question.
- You cannot go back to previous questions.
- There are no wrong answers. It’s about what you think or do.
- At the top of the page, you can see how far along you are.

As a thank-you, we are giving away x number of €25 VVV gift cards! You can use these at, for example, bol.com or Thuisbezorgd.

More information about the study and your privacy can be found and downloaded [here].

*Link to the information sheet*

Have questions or experiencing any problems while filling out the questionnaire? Contact the researchers via email (email blinded).

**Online consent form for the study: How do young people think about condom use?**

I have read and understood the ‘Information sheet for participants’ for this study. I understand that I can stop participating at any time. The researchers will use the data I have provided until that point. I consent to participate in this study.

That means I give permission for:

1. Participation in the study.
2. Collecting data about who I am (gender, age, and background).
3. Collecting data about what I do (condom use and sexual activities).
4. Collecting data about my sexual health (STI tests and diagnoses).

o Yes

o No

I agree that my research data may be stored in Data Archiving and Networked Services (DANS) and on data.rivm.nl, so that other researchers can view them. The data will not reveal who I am. If I do not consent, I can still participate in the study.

o Yes

o No

Now, there are some general questions about you.

1. **How old are you?**

____________________

1. **Were you born as a boy or a girl? (This refers to how your parents registered you with the municipality.)**

- Boy
- Girl

1. **How do you identify? (Your feeling may differ from how you were born.)**

- A boy
- A girl
- Somewhere between a boy and a girl
- Both a boy and a girl
- Neither a boy nor a girl
- Other, namely ...
- I don’t know (yet)

If 2 and 3 indicate that the participant is not cisgender:

1. **Have you had surgery to change your genitalia?**

- Yes, I was born with a vagina and now have a penis.
- No, I was born with a vagina and still have a vagina.
- Yes, I was born with a penis and now have a vagina.
- No, I was born with a penis and still have a penis.

1. **How do you see yourself?**

- Heterosexual
- Homosexual or gay
- Lesbian or gay
- Bisexual
- Pansexual
- Queer
- Asexual
- Other, namely ...
- I don’t know
- I’d rather not say

1. **What is the highest level of education you have completed?**

- Elementary school
- Special education
- VMBO
- MBO 1
- MBO 2, 3, or 4
- HAVO
- VWO
- HBO
- University

1. **In which country were you born?**

- The Netherlands
- Turkey
- Another European country
- Morocco
- Another African country
- Suriname
- The Antilles
- Another South/Central American country
- A North American country
- Indonesia
- Japan
- Another Asian country
- Australia or New Zealand
- I don’t know

1. **In which country was your mother born?**

(Same country options as above)

1. **In which country was your father born?**

(Same country options as above)

1. **With which country or culture do you feel the most connected?**

(Same country options as above)

1. **Is there another country or culture with which you feel connected?**

- Yes
- No

If 11 = Yes:

**12. With which second country or culture do you feel the most connected?**

(Same country options as above)

**Now we’ll ask a few questions about whether you’ve ever had sex.**

1. **Have you ever had vaginal sex (penis in vagina)?**

- Yes
- No
- I’d rather not say

1. **With how many people have you had vaginal sex (penis in vagina) so far?**

Provide an estimate if unsure.

__________________

1. **How old were you when you first had vaginal sex?**

This refers to the first partner you had vaginal sex with after the age of 13. Provide an estimate if unsure.

__________________

**The next questions are about you, your opinions regarding condoms, sex, and health.**

**We will also ask questions about STIs.**

**What is an STI?**

STI stands for sexually transmitted infection. Another word for this is venereal disease.

An example of an STI is chlamydia.

**16. How do you feel about the following statements**?

Strongly Disagree Strongly Agree

1 2 3 4 5

I have a lot of self-confidence.

I trust that I can use a condom.

I trust that I can convince a partner to use a condom.

I plan to use a condom when I have sex.

**17. How do you feel about the following statements?**

Strongly Disagree Strongly Agree

1 2 3 4 5

When I use a condom, I trust that I am protected against STIs.

Using condoms is an effective way to avoid STIs.

When I use a condom, I know for sure that I am protected from STIs.

I would feel bad if I got an STI.

**18. People can get an STI. Thinking about how you live now, how likely do you think it is that you will get an STI?**

Provide a percentage between 0% (absolutely no chance of getting an STI) and 100% (very high chance of getting an STI).

_________________

**19. How do you feel about the following statements?**

Strongly Disagree Strongly Agree

1 2 3 4 5

People who are important to me think I should use a condom when I have sex.

People who are important to me use a condom when they have sex.

I feel comfortable using condoms.

People like me (e.g., classmates and friends) feel comfortable using condoms.

**20. How do you feel about the following statements?**

Strongly Disagree Strongly Agree

1 2 3 4 5

I am motivated to stay healthy.

I think it’s important to use a condom during sex.

I pay less than average attention to my health.

My health is important to me.

I am motivated to protect myself during sex.

I think it’s important to make sure I don’t get an STI.

**21. How do you feel about the following statements?**

Strongly Disagree Strongly Agree

1 2 3 4 5

I use condoms automatically.

I use condoms during sex without consciously remembering to.

I use condoms during sex without thinking about it.

I’m already using a condom before I even realize it.

In the heat of the moment, I often forget to use a condom.

If I use alcohol or drugs, I forget to use a condom.

**22. How do you feel about using condoms?**

Bad Good

1 2 3 4 5

Unpleasant Pleasant

1 2 3 4 5

Not comfortable Comfortable

1 2 3 4 5

Has disadvantages Has advantages

1 2 3 4 5

Irresponsible Responsible

1 2 3 4 5

**The next questions describe a hypothetical scenario. Imagine it is about you and a hypothetical partner.**

Scenario for female:

You are single and you encounter an attractive acquaintance, Fynn, in a bar. You chat for a while and both notice that it's a pleasant conversation. Fynn is funny and genuinely interested in what you have to say. You spend the whole evening together. At the end, he walks you home. You ask if he wants to come in, and he says yes. After chatting and kissing on the couch for a while, it's clear that you both want to have sex with each other. [sentence that varies for each condition]

Scenario for male:

You are single and you encounter an attractive acquaintance, Lynn, in a bar. You chat for a while and both notice that it's a pleasant conversation. Lynn is funny and genuinely interested in what you have to say. You spend the whole evening together. At the end, she walks you home. You ask if she wants to come in, and she says yes. After chatting and kissing on the couch for a while, it's clear that you both want to have sex with each other. [sentence that varies for each condition]

- Attitudes:
  - Positive: Fynn/Lynn says that he/she finds it important and pleasant to use condoms and asks, "what shall we do?"
  - Negative: Fynn/Lynn says that he/she finds it unimportant and unpleasant to use condoms and asks, "what shall we do?"
- Norms:
  - Positive: Fynn/Lynn says that his/her friends have sex with condoms and asks, "what shall we do?
  - Negative: Fynn/Lynn says that his/her friends have sex without condoms and asks, "what shall we do?"
- Risk behavior:
  - Low: Fynn/Lynn has only had one long-term relationship before you and asks, "what shall we do?"
  - High: Fynn/Lynn has had multiple short-term relationships before you and asks, "what shall we do?"

**23. If I were in this situation, I would use a condom.**

Highly unlikely Highly likely

1 2 3 4 5

**24. I think Fynn/Lynn would want to use a condom.**

Highly unlikely Highly likely

1 2 3 4 5

**Now we’ll ask about your sex life and condom use.**

**25. Have you had vaginal sex (penis in vagina) in the past 12 months?**

- Yes
- No
- I’d rather not say

If 25 = No, the survey ends.

**26. In the past 12 months, have you had vaginal sex (penis in vagina)?**

- Yes
- No
- I’d rather not say

END OF SURVEY IF QUESTION 25 = NO

**27. With how many different partners have you had vaginal sex (penis in vagina) in the past 12 months?**

Provide an estimate if you’re not sure.

________________

**27. With how many of these partners did you have sex for the first time in the past 12 months?**

Provide an estimate if you’re not sure.

________________

**28. How often do you use condoms during vaginal sex?**

- Never
- Mostly not
- Sometimes yes, sometimes no
- Mostly yes
- Always

It’s possible that you have engaged in different types of sex in the past 12 months, or that you’ve had sex with someone of the same gender. For the following questions, we are only asking about people with whom you’ve had vaginal sex (penis in vagina).

**29. Partner 1**

Think of the partner you most recently had vaginal sex with. For the following questions, you can use the first letter(s) of their name or a nickname.

First letter(s) or nickname: _________________________

**30. How many times have you had vaginal sex with Partner 1?**

- 1 time
- More than once

If 30 = 1 time:

**31. Did you and Partner 1 use a condom that time?**

- Yes
- No
- I don’t know

If 30 = More than once:

**32. How long did you have vaginal sex with Partner 1?**

- Less than 1 month
- 1 month or longer

**33. How long did you have vaginal sex with Partner 1?**

______ weeks/months

**34. On average, how often did you and Partner 1 have vaginal sex per week/month?**

Provide an estimate if you’re not sure.

________ times per week/month

**35. How often did you and Partner 1 use condoms during the period when you were having sex?**

- Never
- Mostly not
- Sometimes yes, sometimes no
- Mostly yes
- Always

**36. Do you think you and Partner 1 will have sex again in the future?**

- Yes
- No
- I don’t know

*If respondents had ≥ 2 sexual partners in the past 12 months:

The questions about partners (29–35) will be repeated for each partner, up to a maximum of 10 partners. If a respondent reports having more than 1 partner, the same set of questions will be asked for each partner individually.

The survey stops after 5 partners, even if you had 6 or more partners.*

**Now we’ll ask some questions about STIs. After that, the survey is complete.**

**37. Have you ever had an STI test?**

- Yes, in the past 12 months
- Yes, more than 12 months ago
- No

If 37 = Yes:

**38. Have you ever had an STI? If yes, which STI?**

- No, I’ve never had an STI
- Yes, I had chlamydia
- Yes, I had another STI
- Yes, I had chlamydia and another STI
- I don’t know
- If the respondent reports having chlamydia:

**39. When did you have chlamydia?**

- In the past 12 months
- More than 12 months ago
- Both in the past 12 months and before (you’ve had chlamydia multiple times)

**Thank you for completing the questionnaire!**

**Would you like more information about love, sex, or STIs? Visit [sense.info].**

**As a thank-you, you can enter a prize draw. You can win a €25 VVV gift card!**

**With this gift card, you can purchase items at, for example, bol.com or Thuisbezorgd.**

**For the prize draw, we need your email address. The researchers will not see which email address corresponds to which answers.**

**I would like to participate in the prize draw and therefore consent to my email address being used.**

- Yes
- No

| Supplementary Table 1, included TDF domains with underlying constructs and the number of items | | | |
| --- | --- | --- | --- |
|  |  |  |  |
| TDF domain | Construct | Number of items | Items |
| Memory, attention and decision processes | Memory in the heat of the moment | 1 | In the heat of the moment, I often forget to use a condom. |
| Behavioral regulation | Habitual behavior | 4 | 1: Using condoms is something I do automatically. 2: I use condoms during sex without having to consciously remember to do so. 3: I use condoms during sex without having to think about it. 4: I am already using a condom before I notice. |
| Beliefs about capabilities | Self-confidence | 1 | I am self-confident |
|  | Self-efficacy | 1 | I am confident that I can use a condom. |
|  | Negotiation self-efficacy | 1 | I am confident that I can persuade a partner to use a condom. |
| Beliefs about consequences | Risk perception | 1 | People can get an STI. Think about how you live right now. How likely do you think it is that you will get an STI yourself? Provide a percentage between 0% (no chance of getting an STI at all) and 100% (very likely to get an STI) |
|  | Response efficacy | 3 | 1: If I use a condom, I am confident that I am protected against STIs. 2: Condoms are effective in preventing STIs. 3: If I use a condom, I am sure that I'm protected against STIs. |
|  | Perceived severity | 1 | I would be upset if I got an STI. |
| Intention | Intentions | 1 |  |
| Goals | Health goals |  |  |
|  | General health goals | 3 | 1: I am motivated to stay healthy. 2: I am less than average concerned about my health. 3: My health is important to me. |
|  | Sexual health goals | 3 | 1: It is important to me to use a condom during sex. 2: I am motivated to protect myself during sex. 3:It is important to me to make sure I don't get an STI. |
| Emotion | Affective associations |  |  |
|  | Overall | 1 | How do you feel about condoms? Bad/Good |
|  | Affective | 2 | How do you feel about condoms? 1: Pleasant/unpleasant. 2: Enjoyable/unenjoyable. |
|  | Instrumental | 2 | How do you feel about condoms? 1: Harmful/beneficial. 2: Foolish/wise. |
| Environmental context and resources | Memory during intoxication | 1 | When I use alcohol/drug, I forget to use a condom. |
| Social inluences | Normative influences |  |  |
|  | Injunctive normative influences | 1 | People who are important to me think I should use a condom when I have sex. |
|  | Descriptive normative influences | 1 | People who are important to me use a condom when they have sex. |

Supplementary Figures

Figure S1, simple slopes of the interaction between the attitudes condition and condom use intentions.


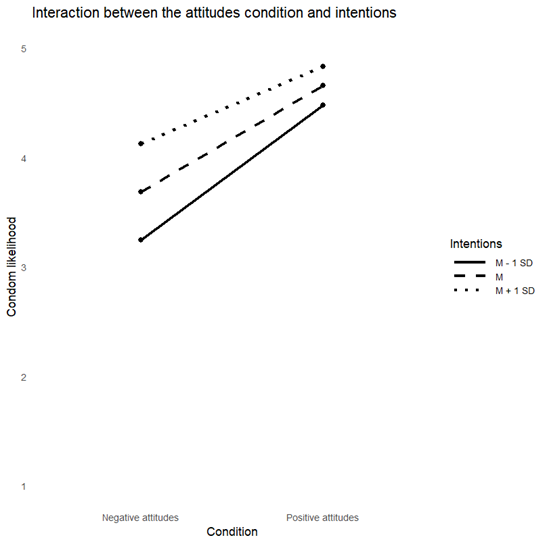


Figure S2, simple slopes of the interaction between the attitudes condition and memory to use a condom.


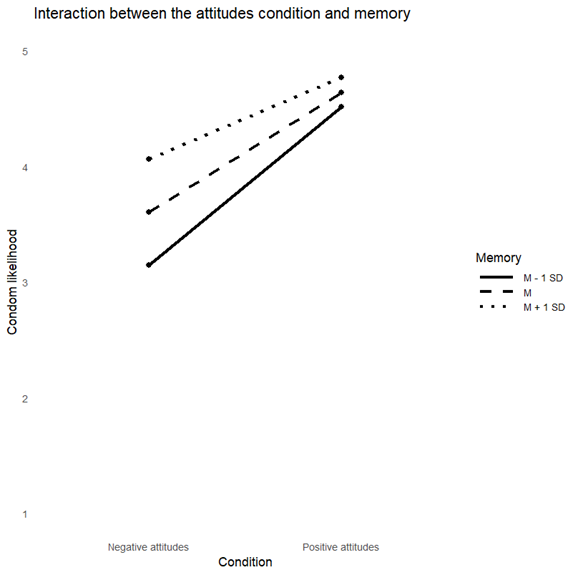


Figure S3, simple slopes of the interaction between the attitudes condition and memory to use a condom during alcohol/drug use.


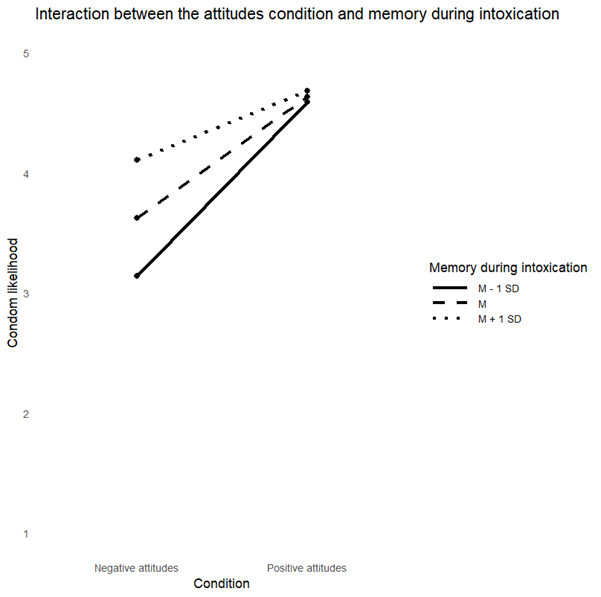


Figure S4, simple slopes of the interaction between the attitudes condition and condom use habitual behavior.


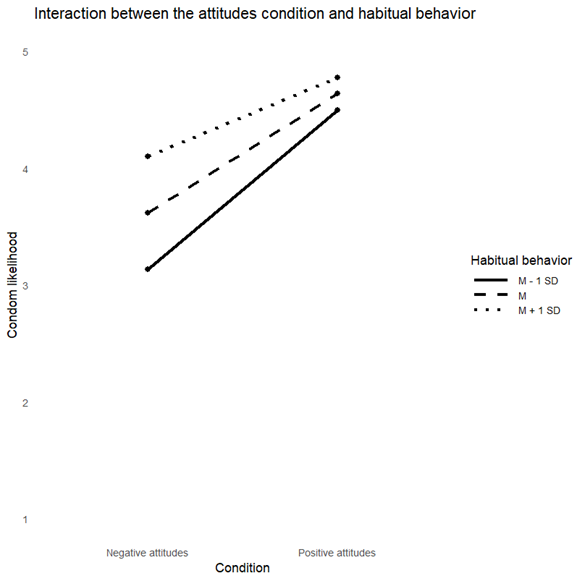


Figure S5, simple slopes of the interaction between the attitudes condition and affective associations.


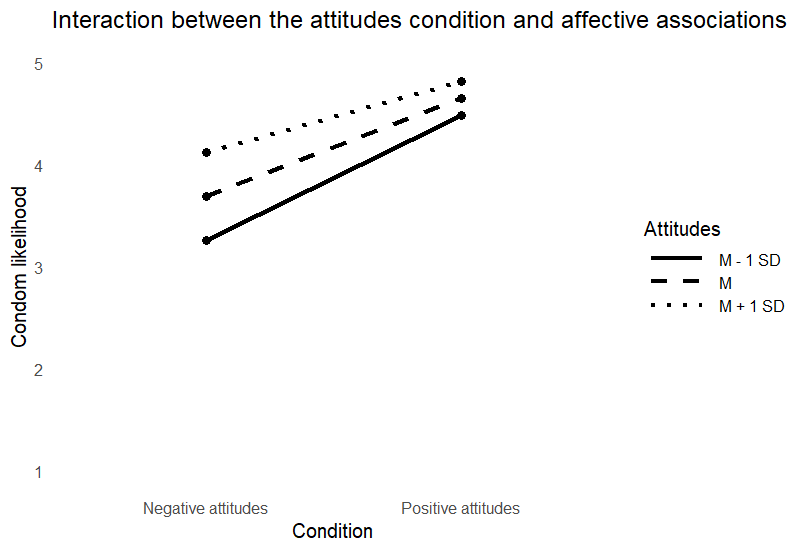


Figure S6, simple slopes of the interaction between the attitudes condition and the number of lifetime partners. *Due to a large standard deviation, the value of M - 1 SD is negative.


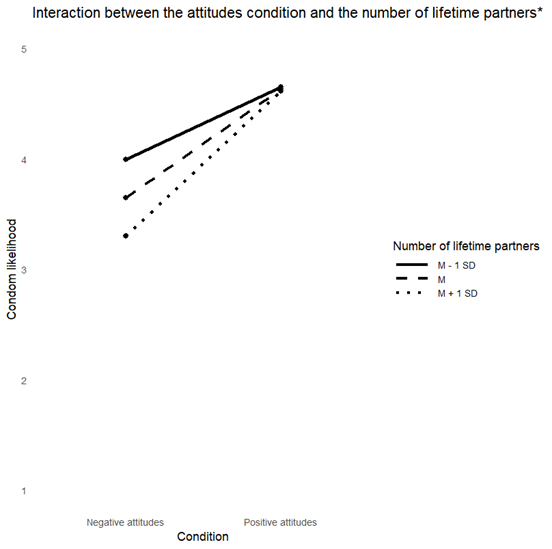


Figure S7, simple slopes of the interaction between the attitudes condition and the number of recent partners. * Due to a large standard deviation, the value of M - 1 SD is negative.


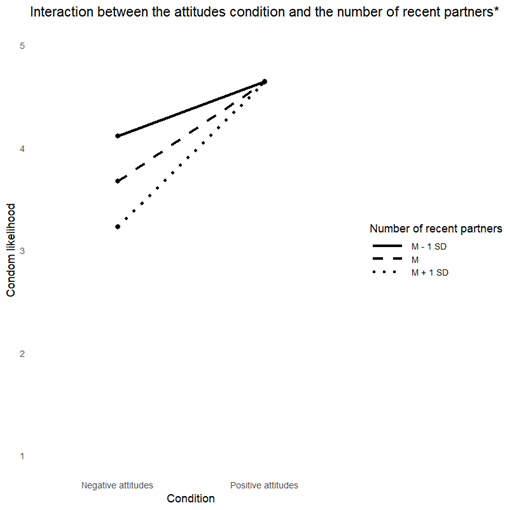


Figure S8, simple slopes of the interaction between the attitudes condition and condom response efficacy.


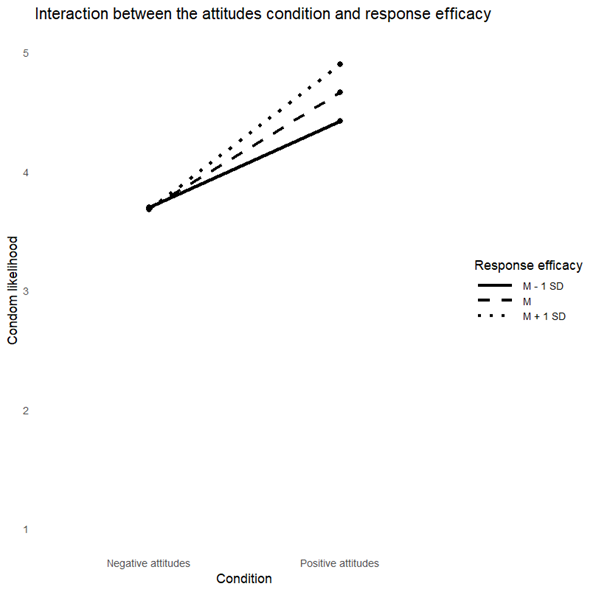


Figure S9, simple slopes of the interaction between the norms condition and the number of lifetime partners. * Due to a large standard deviation, the value of M - 1 SD is negative.


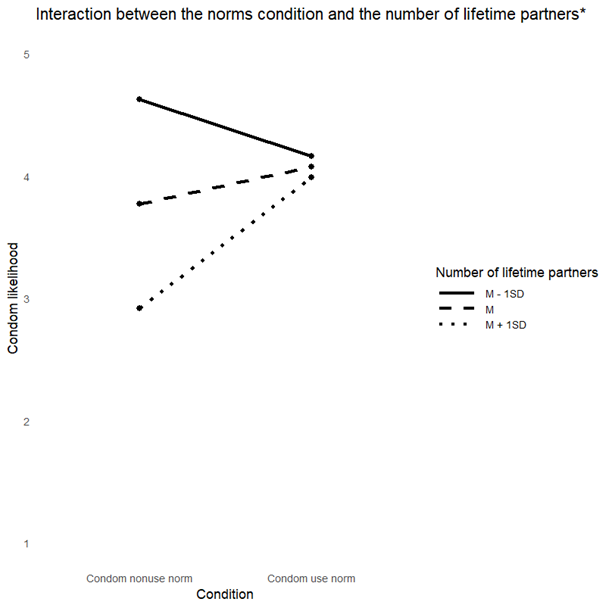


Figure S10, simple slopes of the interaction between norms condition and descriptive normative influences.


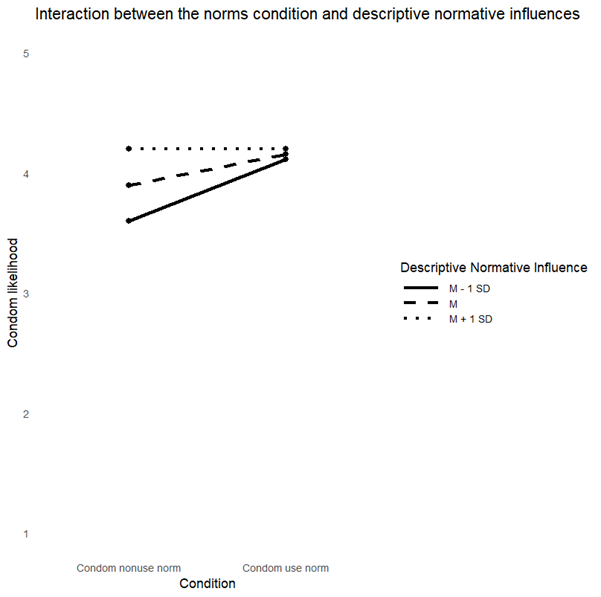


Figure S11, simple slopes of the interaction between the norms condition and affective associations.


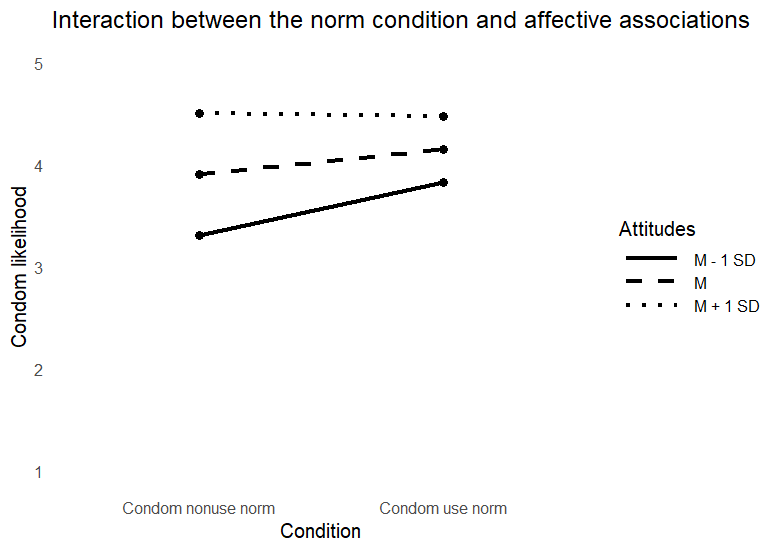


Figure S12, simple slopes of the interaction between the risk behavior condition and memory to use a condom.
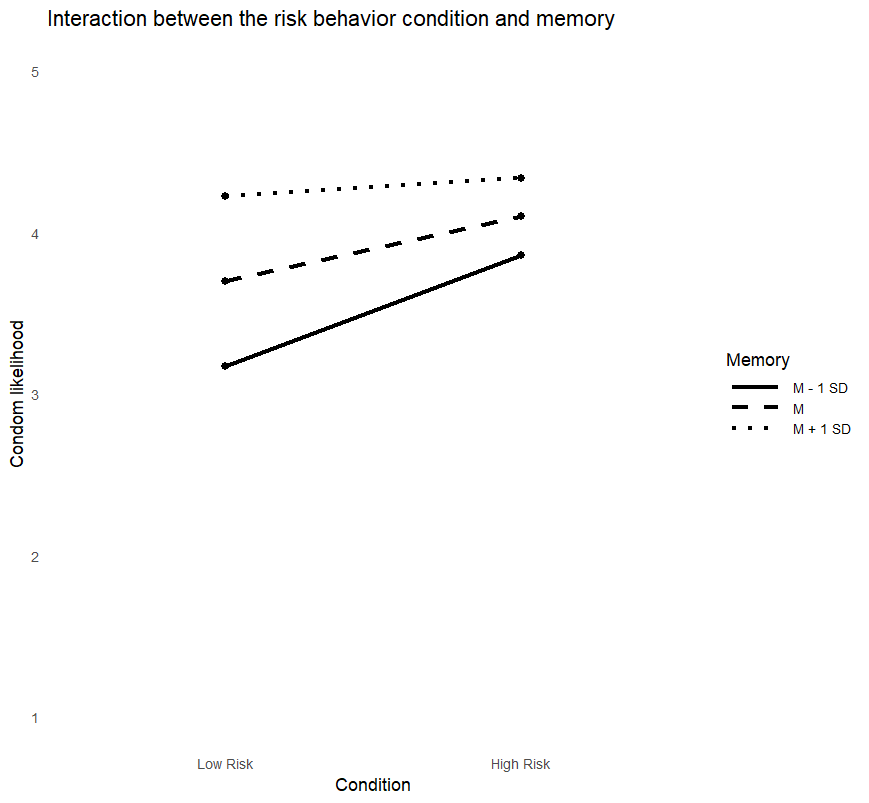


Figure S13, simple slopes of the interaction between the risk behavior condition and attitudes.


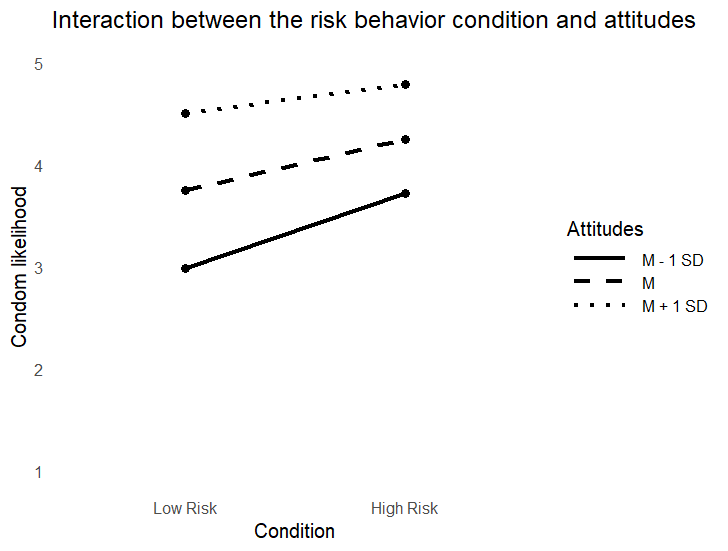


Figure S14, simple slopes of the interaction between the risk behavior condition and the number of lifetime partners. *Due to a large standard deviation, the value of M - 1 SD is negative
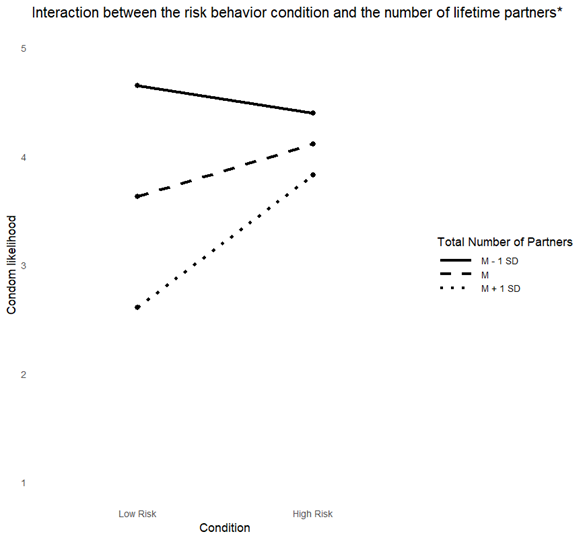

Supplement: Supplementary file 1 — Supplementary Material 1. [file 12889_2025_24741_MOESM1_ESM.docx]
